# Real-Time Transient Voltage and Frequency Sensing Strategy for Resilience Enhancement of PV-Storage Systems in Weak Grids

**DOI:** 10.3390/s26113412

**Published:** 2026-05-28

**Authors:** Yu Ji, Zixuan Liu, Xin Gu, Chenze Huo, Zihan Zhang, Song Tang, Jun Mei, Can Huang

**Affiliations:** 1 School of Electric Power Engineering, Nanjing Institute of Technology, Nanjing 211167, China; y00450240521@njit.edu.cn (Z.L.); y00450240815@njit.edu.cn (X.G.); x00206241315@njit.edu.cn (C.H.); x00206251014@njit.edu.cn (Z.Z.); tangsong@njit.edu.cn (S.T.); 2 School of Electrical Engineering, Southeast University, Nanjing 210096, China; mei_jun@seu.edu.cn (J.M.); canhuang@seu.edu.cn (C.H.)

**Keywords:** transient-state sensing, signal processing, weak grids, photovoltaic-storage system, hybrid energy storage system, virtual synchronous generator, adaptive virtual impedance, measurement noise immunity

## Abstract

Photovoltaic (PV)-storage systems operating in weak grids are affected by high grid impedance, transient voltage disturbances, and measurement noise, which can degrade frequency regulation, increase converter current stress, and impose high-frequency current fluctuations on the battery. To address these issues, this paper proposes a multi-timescale transient-state sensing and signal-processing framework for grid-forming PV-hybrid storage systems. The proposed framework combines three coordinated functions. First, a frequency-domain HESS power-decoupling mechanism separates high-frequency transient power components and assigns them to the supercapacitor, while the battery mainly handles low-frequency energy variations. Second, a voltage-deviation-driven adaptive virtual inductance is introduced to increase the equivalent output impedance during voltage-sag events and reduce transient inrush current. Third, a noise-resilient frequency sensing strategy based on a filtered frequency derivative and a dead-band for false-trigger suppression is developed to reduce noise-induced false triggering in adaptive inertia and damping control. Comparative simulations indicate that under the tested weak-grid conditions, the proposed method reduces the transient inrush-current peak by 53.2%, decreases the maximum dynamic frequency deviation by approximately 75%, and improves the active-power regulation speed by more than 50%. These results indicate that the proposed sensing-oriented framework can improve transient response while reducing converter and battery current stress in PV-storage systems connected to high-impedance grids.

## 1. Introduction

Driven by the global imperative to achieve carbon neutrality and facilitate sustainable energy transitions, the large-scale integration of distributed renewable energy sources, particularly photovoltaic (PV) generation, has accelerated unprecedentedly [1]. However, the intrinsic intermittency and stochasticity of solar irradiance pose severe challenges to real-time power balance, driving the urgent need for advanced grid-forming technologies and centralized volatility reduction mechanisms [2,3]. To mitigate these fluctuations, hybrid PV-energy storage integration has emerged as an important infrastructure. Through the bidirectional energy buffering of storage units, PV-storage systems convert intermittent renewable generation into dispatchable power, providing essential support for future low-inertia grids [4]. In addition to generation intermittency, the allocation and penetration level of PV plants in distribution networks can also affect voltage profiles, reverse power flow, power quality, and system stability. Mansouri et al. reviewed these impacts and the corresponding solutions for PV plants in distribution networks [5]. This paper focuses on improving the transient sensing and grid-forming control capability of a PV-storage unit under weak-grid conditions, while network-level PV-storage allocation will be considered in future work.

Simultaneously, the widespread replacement of conventional synchronous machines with power electronic converters has substantially reduced the physical inertia and damping of the power system [6]. This transition has led to the emergence of weak grids, characterized by high line impedance [7]. Under such high-impedance conditions, traditional grid-following inverters exhibit reduced stability during transient disturbances, frequently resulting in severe power-frequency oscillations [8,9]. To mitigate these stability issues, the Virtual Synchronous Generator (VSG) control strategy has been extensively investigated [10]. By emulating the electromechanical swing equations and excitation characteristics of synchronous machines, grid-forming VSGs provide converters with virtual inertia and damping, thereby enhancing the frequency and voltage support capabilities of renewable energy plants [11].

Despite its theoretical advantages, the practical application of conventional VSG control in weak grids is limited by its fixed-parameter design [12]. Recent studies demonstrate that an increase in grid impedance degrades the transient power transfer capability, resulting in deteriorated dynamic performance. This impedance coupling lowers the active power transmission limit, manifesting as a “transient power transfer restriction” and “dynamic hysteresis.” Consequently, the system experiences sluggish active power tracking and prolonged secondary oscillations during grid disturbances, undermining its operational resilience. Fundamentally, these dynamic constraints are exacerbated by the challenges of accurate state sensing in high-impedance environments, where traditional phase-locked loops (PLLs) and measurement units are highly susceptible to background noise and transient signal distortion [13].

To mitigate these dynamic constraints, extensive studies have investigated adaptive control strategies for grid-forming converters. Single-parameter adaptive methods adjust either virtual inertia or damping to limit the rate of change of frequency or attenuate electromechanical oscillations [14,15]. Dual-parameter and coordinated damping strategies further improve the compromise between transient overshoot and settling speed [16,17,18]. More recently, data-driven and reinforcement-learning-based impedance emulation methods have also been explored to improve overcurrent suppression and dynamic adaptability [19]. These studies have significantly advanced the dynamic regulation capability of VSG-controlled converters. However, most of them focus primarily on the electromechanical power-frequency response, while the sensing process that provides the frequency derivative, voltage deviation, and disturbance classification signals is often assumed to be ideal. From the perspective of transient sensing and signal processing, this assumption may become restrictive in weak-grid PV-storage converters, because sensor noise, sampling delay, PLL-induced frequency-estimation errors, and derivative amplification can distort RoCoF and PCC voltage-deviation information, leading to false triggering or delayed activation of adaptive control loops.

Recently, synergistic adaptive strategies, such as the exponential coordination of virtual inertia and damping proposed by the authors in [20], have been developed to mitigate overdamped hysteresis in high-impedance grids. However, relying solely on electromechanical parameter tuning still leaves several practical issues unresolved. First, frequency-derivative-based adaptive control depends on reliable transient frequency sensing. In practical digital controllers, measurement noise, sampling delay, and derivative amplification may affect the reliability of RoCoF estimation and trigger unnecessary high-frequency adaptive actions if no filtering or dead-band logic is used [21,22]. Second, severe voltage-sag faults may induce electromagnetic-timescale inrush currents before the outer power-frequency loop can respond. Although current-limiting and virtual-impedance-based strategies have been widely investigated for grid-forming converters under low-voltage ride-through and fault conditions, fixed or improperly tuned virtual impedance may still introduce a compromise between steady-state voltage regulation, transient current suppression, and synchronization stability [23,24,25]. Third, aggressive power tracking may impose high-frequency transient current stress on the DC link and the storage system. Although HESS-based smoothing methods can allocate low-frequency power variations to batteries and high-frequency components to supercapacitors, most studies treat HESS mainly as a DC-side energy management problem, and its coordination with weak-grid transient sensing and grid-forming control remains insufficiently clarified [26,27,28]. Therefore, the core bottleneck is not only mathematical power tracking but also the reliable detection, filtering, and classification of transient grid states under noisy weak-grid conditions.

Existing studies have improved weak-grid PV-storage operation from different perspectives, but several coupled transient problems remain insufficiently addressed. Adaptive VSG methods can improve electromechanical power-frequency dynamics by tuning virtual inertia and damping, but they usually assume reliable frequency-derivative measurements and do not explicitly address noise-induced false triggering. Virtual-impedance and current-limiting methods can suppress fault current during voltage sags, but they are generally designed for electromagnetic-timescale protection and are not coordinated with adaptive frequency regulation or DC-side storage stress mitigation. HESS smoothing methods can reduce PV power fluctuation on the DC side, but they are usually decoupled from weak-grid transient sensing and grid-forming control. Therefore, existing single-function methods still experience difficulty in simultaneously handling voltage-sag inrush current, noisy frequency-derivative sensing, weak-grid power-frequency dynamics, and high-frequency battery-current stress within one coordinated framework.

To overcome these limitations, this paper proposes a multi-timescale transient-state sensing and signal-processing framework for weak-grid PV-HESS grid-forming systems. The key idea is to classify transient disturbances according to their dominant physical timescales and map them to the corresponding control or storage response. Specifically, PCC voltage deviation is used to activate adaptive virtual inductance for electromagnetic-timescale inrush-current suppression; the filtered frequency derivative and dead-band logic are used to adjust virtual inertia and damping for electromechanical-timescale frequency regulation while reducing noise-induced false triggering; and frequency-domain HESS power decoupling is used to assign high-frequency transient power components to the supercapacitor, thereby reducing the direct exposure of the battery to high-frequency current stress.

Compared with the authors’ previous work [20], which mainly focused on the electromechanical coordination of virtual inertia and damping, the present work extends the control objective from power-frequency regulation to multi-timescale transient sensing and hardware-stress reduction. The proposed method therefore differs from a simple combination of adaptive VSG, virtual impedance, and LPF-based HESS smoothing. Its main improvement lies in coordinating these functions through a unified sensing-to-action mechanism, so that different weak-grid transients are detected, filtered, and handled by the most suitable control or energy-storage element.

The main contributions of this paper are summarized as follows:

(1) A multi-timescale transient-state sensing framework is proposed for weak-grid PV-HESS grid-forming systems. Unlike conventional adaptive VSG methods that mainly regulate electromechanical power-frequency dynamics, the proposed framework classifies weak-grid disturbances according to their dominant physical timescales and maps them to different control actions, including electromagnetic-timescale current suppression, electromechanical-timescale frequency regulation, and DC-side storage power buffering.
(2) A coordinated voltage/frequency sensing-based adaptive control strategy is developed to address transient overcurrent and noise-induced false triggering. The PCC voltage deviation is used to activate adaptive virtual inductance for voltage-sag inrush-current suppression, while the filtered frequency derivative and dead-band logic are used to regulate virtual inertia and damping without repeatedly triggering the adaptive loop under measurement noise.
(3) A frequency-domain HESS power-decoupling mechanism is integrated with the grid-forming control framework to reduce battery exposure to high-frequency transient power. Different from conventional HESS smoothing methods that are usually treated as DC-side energy management alone, the proposed design coordinates HESS power allocation with weak-grid transient sensing so that fast power fluctuations are mainly assigned to the supercapacitor, while the battery handles slower energy variations.

The remainder of this paper is organized as follows. Section 2 introduces the PV-storage VSG architecture and the HESS energy decoupling strategy. Section 3 analyzes the physical limitations in high-impedance weak grids, focusing on transient overcurrents and power transfer restrictions. Section 4 details the proposed multi-timescale transient-state sensing and signal-processing framework, including parameter optimization constraints. Section 5 presents the comparative simulation results and discussion, including quantitative performance evaluation and practical limitations. Finally, Section 6 concludes the paper and outlines future research directions.

## 2. System Architecture and Sustainable Energy Management Framework

**Figure 1:**
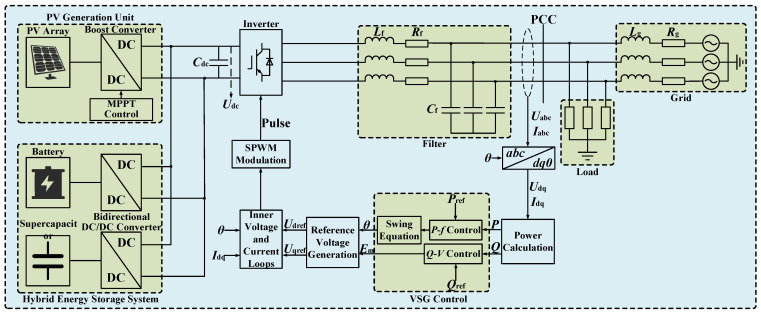
Overall topology of the resilient grid-forming photovoltaic (PV)-storage system with hybrid energy management.

The overall topology of the proposed resilient photovoltaic (PV)-storage grid-connected system is illustrated in Figure 1. On the DC side, the system comprises a PV generation unit and a Hybrid Energy Storage System (HESS). The DC side acts as a sustainable energy buffer, while the DC/AC inverter on the AC side interfaces with the utility grid through an LCL filter. The inverter is governed by the Virtual Synchronous Generator control architecture to provide transient resilience and grid-forming capabilities.

When operating in grid-connected mode, the power balance equation of the system must satisfy the following dynamic constraint:

(1) $$P_{\mathrm{PV}}+P_{\mathrm{bat}}+P_{\mathrm{sc}}-P_{s}=P_{e}=P_{\mathrm{load}}+P_{\mathrm{grid}}$$

where *P*_PV_, *P*_bat_, and *P*_sc_ are the active powers of the PV array, battery, and supercapacitor, respectively; *P*_s_ represents the system power losses; *P*_e_ is the inverter’s electromagnetic output power; *P*_load_ is the local load power; and *P*_grid_ is the injected grid power.

### 2.1. Frequency-Domain Signal Extraction and Storage Buffering Mechanism

**Figure 2:**
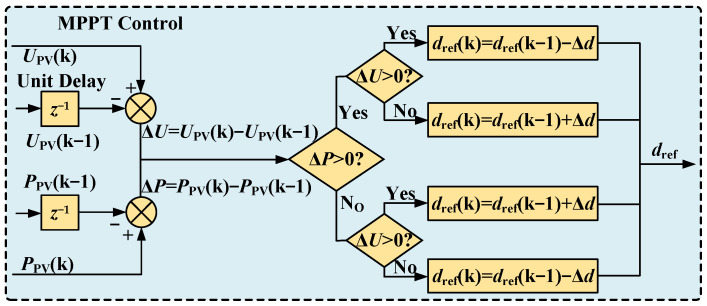
Detailed control block diagram of the P&O-based MPPT algorithm for the PV array.

The PV generation unit interfaces with the DC bus via a DC/DC boost converter, employing the Perturb and Observe (P&O) Maximum Power Point Tracking (MPPT) algorithm to maximize solar energy harvesting, as shown in Figure 2.

As illustrated in Figure 2, the P&O algorithm continuously samples the PV output voltage and power, comparing them with previous states to determine the power gradient. Based on the sign of this gradient (Δ*P* and Δ*U*), it dynamically adjusts the duty cycle reference (*d*_ref_), with a fixed step size to effectively match the equivalent input impedance of the converter with the internal resistance of the PV array. This closed-loop tracking helps the PV system operate near its maximum power point under varying irradiance conditions, providing a reliable and sustainable primary energy source for the subsequent grid-forming Virtual Synchronous Generator.

However, the intrinsic stochasticity and intermittency of solar irradiance cause severe power fluctuations. If these raw fluctuations are directly compensated by a conventional single-battery storage system, the battery may experience frequent high-C-rate charge/discharge cycles, which can accelerate capacity degradation and increase replacement costs [29].

**Figure 3:**
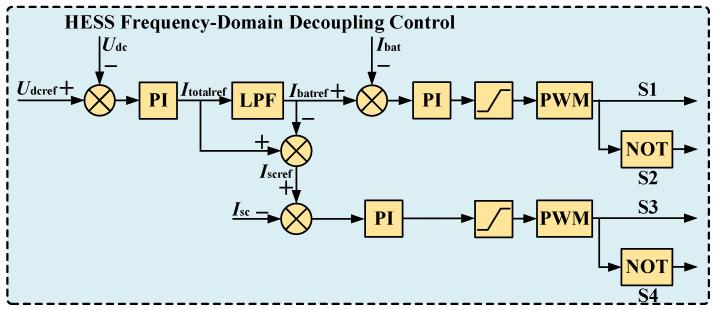
Control block diagram of the sustainable Hybrid Energy Storage System (HESS) based on frequency-domain power decoupling.

To improve the operational sustainability and reduce the high-frequency stress of the energy storage assets, a Hybrid Energy Storage System (HESS) utilizing a frequency-domain power decoupling strategy is adopted, as depicted in Figure 3.

The supercapacitor, characterized by its high power density, is assigned to autonomously absorb the high-frequency transient power spikes. Meanwhile, the battery, possessing high energy density, smoothly handles the low-frequency steady-state energy shifts. By employing a first-order Low-Pass Filter (LPF) as the core signal processing unit, the reference current for the battery (*I*_batref_) is effectively isolated from high-frequency measurement noise and transient power ripples:

(2) $$I_{\mathrm{batref}}=\frac{1}{1+\tau s}I_{\mathrm{totalref}}$$

where *τ* is the filter time constant defining the frequency boundary. Through this mechanism, the transient high-frequency fluctuating components, which are associated with increased battery current ripple and possible micro-cycling stress, are redirected to and absorbed by the supercapacitor unit [29]. Consequently, only the smoothed, low-frequency energy demand is dispatched to the battery as its actual tracking command. This active decoupling architecture serves as an effective physical buffer for the energy storage system. By diverting part of the high-frequency fluctuating power to the supercapacitor, the proposed mechanism reduces the high-frequency current stress imposed on the battery, which is beneficial for improving the operating conditions of the storage system.

### 2.2. Grid-Forming Resilience Interface Based on VSG

**Figure 4:**
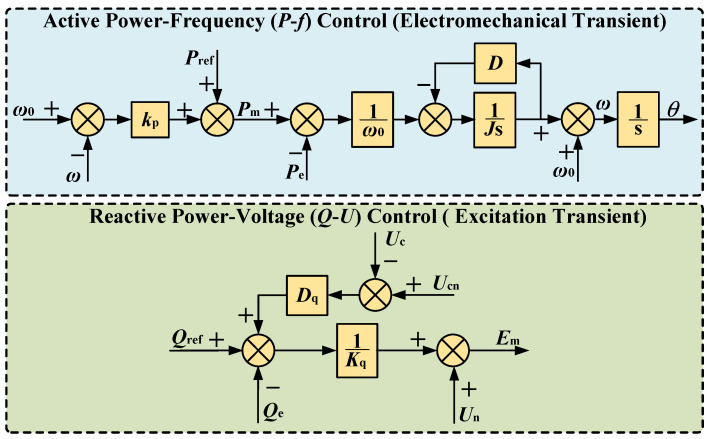
Overall block diagram of the grid-forming VSG with embedded transient sensing and signal processing loops.

To provide inertia and damping support to the weak grid and mitigate severe transient deviations during disturbances, the inverter utilizes a grid-forming VSG architecture. As comprehensively illustrated in the VSG control block diagram in Figure 4, the framework consists of two coupled regulating loops.

The active power-frequency (*P*-*f*) control loop mimics the swing equation of a synchronous machine to provide continuous electromechanical resilience:

(3) $$J\frac{d\omega }{dt}=T_{m}-T_{e}-T_{D}=T_{m}-T_{e}-D(\omega -\omega_{0})$$

where *J* is the virtual inertia; *D* is the damping coefficient; *ω* is the actual angular speed of the system; *T*_m_, *T*_e_, and *T*_D_ are the mechanical, electromagnetic, and damping torques, respectively.

Based on the relationship between torque and power, the above equation can be rewritten as

(4) $$\frac{P_{\mathrm{ref}}+k_{p}(\omega_{0}-\omega )-P_{e}}{\omega_{0}}-D(\omega -\omega_{0})=J\frac{d\omega }{dt}$$

where *ω*_0_ is the rated angular speed; *P*_ref_ is the reference output active powers; *k*_p_ is the active power droop coefficient.

Simultaneously, the reactive power-voltage (*Q*-*U*) control loop emulates the excitation system to regulate the output voltage amplitude (*E*_m_):

(5) $$E_{m}=U_{n}+\frac{1}{K_{q}}\left[\left(Q_{\mathrm{ref}}-Q_{e})+D_{q}(U_{\mathrm{cn}}-U_{c}\right)\right]$$

where *Q*_ref_ and *Q*_e_ are the reference and measured reactive powers, respectively; *D*_q_ is the reactive-power damping coefficient; *U*_cn_ is the rated terminal voltage; *U*_c_ is the measured terminal voltage; *U*_n_ is the rated grid-voltage magnitude; *K*_q_ is the integrator coefficient of the reactive–voltage loop; and *E*_m_ is the voltage magnitude generated by the reactive–voltage control loop.

Rather than adopting rigid Phase-Locked Loops (PLLs) that are prone to instability in weak grids, this coupled electromechanical emulation grants the inverter an inherent frequency and voltage buffering capability. This control structure helps improve the frequency and voltage support capability of the converter under weak-grid conditions.

### 2.3. Virtual-Impedance-Based Current-Limiting Mechanism for Converter Protection

Beyond energy management, improving the current-limiting capability of the converter under severe voltage-sag events is important for system resilience. Semiconductor power devices, such as IGBTs, are sensitive to transient overcurrent stress during fault conditions [30]. To provide an impedance-based current-limiting mechanism, a virtual impedance *Z*_vir_(s) is embedded within the voltage control loop. The equivalent output reference voltage U^*^_ref_ is reshaped as

(6) $$U_{\mathrm{ref}}^{*}=E_{m}-Z_{\mathrm{vir}}\left(s\right)\cdot I_{o}$$

where *I*_o_ is the inverter output current vector.

In the d-q synchronous rotating reference frame, to achieve decoupled control of active and reactive power, the virtual impedance typically comprises a resistance component *R*_vir_ and an inductance component *L*_vir_. Fully considering the cross-coupling terms introduced by the coordinate transformation, its d–q axis components can be expanded as

(7) $$\left\{\begin{array}{l} U_{d}^{*}=U_{d}-\left(R_{\mathrm{vir}}I_{d}-\omega_{0}L_{\mathrm{vir}}I_{q}\right)\\ U_{q}^{*}=U_{q}-\left(R_{\mathrm{vir}}I_{q}+\omega_{0}L_{\mathrm{vir}}I_{d}\right) \end{array}\right.$$

where *U*_d_ and *U*_q_ are the d–q axis components of the original reference voltage; *I*_d_ and *I*_q_ are the d–q axis components of the output current.

By introducing the virtual inductance *L*_vir_, the inductive component of the system is effectively increased, making the total output impedance highly inductive, which satisfies the prerequisite for decoupled power control in VSGs. Given that the independent regulation of active and reactive power in VSGs relies on a highly inductive output impedance, and the grid impedance in a weak grid environment is predominantly inductive, this paper neglects the effect of virtual resistance (i.e., setting *R*_vir_ = 0) to simplify the control system design and ensure power decoupling; the system can increase its equivalent output impedance during detected fault conditions. This provides an additional impedance-based current-limiting effect, which helps reduce the short-circuit inrush current and mitigate converter overcurrent stress under severe operating conditions.

## 3. Resilience Bottlenecks in Weak Grids: Transient Overcurrent and Power Transfer Restrictions

### 3.1. Vulnerability of Power Electronics to Transient Inrush Currents

Given the assumption that the virtual resistance is neglected (*R*_vir_ = 0) to ensure decoupled active and reactive power control, the fundamental relationship between the inverter output voltage *U*_o_ and the PCC voltage *U*_pcc_ can be expressed as

(8) $$U_{o}=U_{\mathrm{pcc}}+j\omega L_{\mathrm{vir}}I_{o}+j\omega L_{g}I_{o}$$

where *L*_g_ is the grid inductance. During steady-state operation, the voltage drop Δ*U*_vir_ induced by the virtual impedance can be approximately quantified using the longitudinal component of the voltage drop:

(9) $$\Delta U_{\mathrm{vir}}=\frac{PR_{\mathrm{vir}}+QX_{\mathrm{vir}}}{U_{\mathrm{cn}}}=\frac{Q\omega L_{\mathrm{vir}}}{U_{\mathrm{cn}}}$$

Equation (9) indicates that the voltage drop generated by the virtual inductance is approximately proportional to both the load reactive power *Q* and the virtual inductance *L*_vir_. Consequently, under heavy or highly inductive load conditions, an excessively large *L*_vir_ may cause a noticeable PCC voltage drop. This voltage drop can degrade power quality and may cause the PCC voltage to approach or fall below the standard lower limit.

Conversely, when a sudden grid voltage sag Δ*U* occurs, a significant transient inrush current Δ*I*_surge_ is generated before the outer control loops can react. This transient inrush current is limited primarily by the total physical and virtual impedance of the system:

(10) $$\Delta I_{\mathrm{surge}}=\frac{\Delta U}{\omega \left(L_{\mathrm{line}}+L_{g}+L_{\mathrm{vir}}\right)}$$

Equation (10) indicates that the amplitude of the transient surge current is inversely proportional to the total equivalent inductance. In a conventional VSG with a fixed or zero *L*_vir_, the physical grid inductance (*L*_g_) alone is often insufficient to clamp the instantaneous overcurrent. This unmitigated transient current exposes the inverter to excessive thermal stress on the insulated-gate bipolar transistors (IGBTs), potentially leading to system tripping [30].

**Figure 5:**
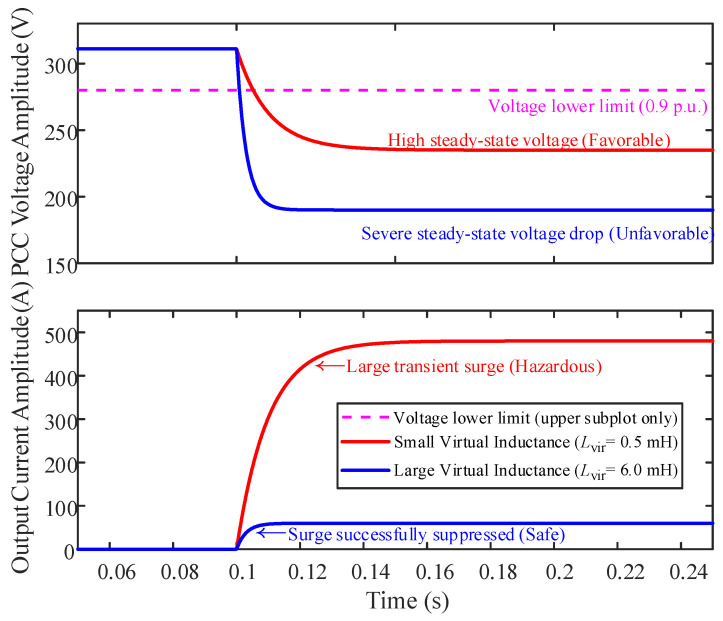
Comparison of step responses under different virtual inductances *L*_vir_.

To illustrate this coupling effect, the dynamic step responses of the system under different virtual inductance values are compared in Figure 5.

As illustrated in Figure 5, tuning a fixed virtual inductance *L*_vir_ involves a fundamental trade-off between steady-state voltage accuracy and transient overcurrent suppression. Specifically, a small *L*_vir_ (e.g., 0.5 mH) maintains an excellent steady-state voltage well above the lower limit, but it fails to prevent a large transient current spike during a transient fault. Conversely, a large *L*_vir_ (e.g., 6 mH) effectively reduces the surge current under the tested condition, but it significantly degrades the steady-state PCC voltage, potentially violating grid integration codes.

Therefore, conventional fixed-parameter control strategies are insufficient for complex weak grid conditions. This limitation motivates the development of a voltage-deviation-driven adaptive virtual inductance strategy. By keeping *L*_vir_ minimized during steady-state operations to ensure voltage stability and dynamically increasing it during transient disturbances to suppress overcurrents, this approach achieves a coordinated optimization of both steady-state and dynamic performance.

### 3.2. Degradation of Physical Resilience: Power Transfer Restriction and Hardware Fatigue

Beyond hardware thermal limits, the high impedance of weak grids degrades the transient power transfer capability of the system. The electromechanical swing characteristics of a grid-connected VSG are inherently coupled with the grid impedance. From a hardware reliability perspective, this impedance coupling primarily manifests as a significant restriction on transient power transfer.

The instantaneous active power transfer *P*_e_ between the inverter and the grid is strictly constrained by the total equivalent reactance *X* (which is dominated by the grid inductance *L*_g_ in a weak grid environment), governed by the classical power-angle relationship:

(11) $$P_{e}=\frac{U_{o}U_{g}}{X}\sin\delta$$

where *U*_o_ and *U*_g_ are the voltage amplitudes of the inverter output and the grid, respectively, and *δ* is the power angle. The theoretical active power transmission limit of the system is thus defined as

(12) $$P_{\max}=\frac{U_{o}U_{g}}{X}$$

**Figure 6:**
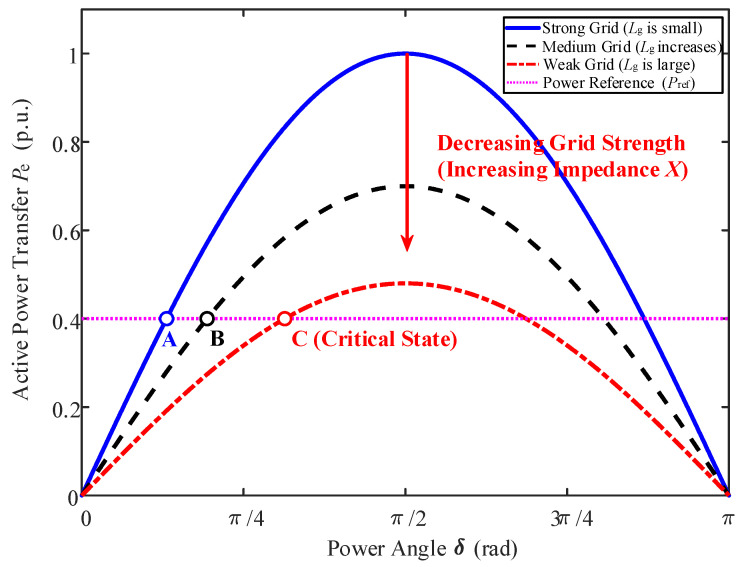
Active power–angle characteristic curves and the degradation of transient energy transfer limits under varying grid strengths.

To illustrate this physical limitation, the active power–angle characteristic curves under varying grid strengths are plotted in Figure 6.

When operating in a nominal strong grid, *L*_g_ is relatively small, yielding a relatively high *P*_max_. The system operates at Point A to deliver the steady-state power reference *P*_ref_, while maintaining a sufficient transient power margin. However, as the grid integration point weakens and *L*_g_ increases, the total reactance *X* increases correspondingly. As indicated by the downward arrow, this increase in impedance lowers the active-power transmission ceiling.

Consequently, to output the same *P*_ref_, the steady-state operating point shifts toward a larger power angle, moving from Point A to Point B and eventually to Point C. At Point C, the system operates near the transmission limit, where the transient power margin becomes very small.

This depleted margin creates a pronounced physical restriction during grid disturbances or load steps. When the virtual rotor of the VSG attempts to release or absorb kinetic energy to provide transient inertia support, the compressed transmission ceiling physically blocks this instantaneous AC power exchange. As a result, this part of the restricted transient power may be reflected to the DC link, resulting in DC-link voltage fluctuations. For a conventional single-battery PV-storage system, the inability of traditional sensing mechanisms to rapidly detect and isolate these transient grid faults means that the high-frequency reflected power translates directly into severe DC bus voltage ripples and large transient energy excursions on the DC side.

Subjecting the battery to unmitigated high-frequency power transients may increase current ripple, charge/discharge reversals, and micro-cycling stress, which are closely related to battery aging and thermal loading. Therefore, conventional fixed-parameter tuning alone is insufficient to reduce DC-side storage stress under weak-grid disturbances. A multi-timescale resilient framework is required to coordinate AC-side transient control with DC-side HESS power decoupling. In this paper, the HESS is used to divert high-frequency transient power components to the supercapacitor, thereby reducing measurable battery-current stress indicators. It should be noted that this work does not claim a complete electrochemical lifetime prediction; instead, the battery-stress mitigation effect is evaluated using current-based indicators in the simulation section.

### 3.3. Multi-Timescale Adaptive Tuning Principles for Resilience Enhancement

**Figure 7:**
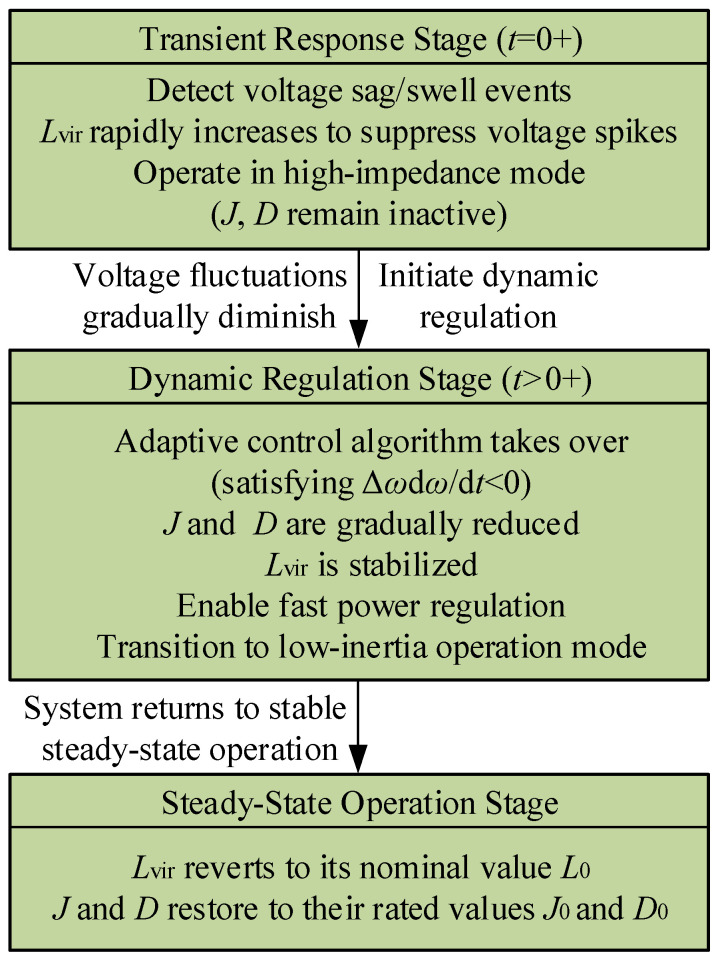
Flowchart of the multi-timescale synergistic sensing and operational logic for the proposed adaptive framework.

To address the aforementioned overcurrent risks and overdamped hysteresis, the VSG control parameters (*L*_vir_, *J*, *D*) must be dynamically coordinated. Instead of a rigid fixed-parameter approach, this paper proposes a multi-timescale operation logic based on the physical progression of grid disturbances. The complete logical framework of this stage-based adaptation is systematically illustrated in Figure 7.

As depicted in the initial stage of Figure 7 (*t* = 0^+^), when triggered by grid events such as voltage sags or swells, the system promptly detects the abrupt voltage mutation. During the initial electromagnetic transient phase, the virtual inductance *L*_vir_ increases rapidly to suppress potential voltage and inrush current spikes. Meanwhile, constrained by the system’s inherent electrical inertia, the virtual inertia *J* and damping coefficient *D* remain inactive. Consequently, the converter operates in a high-impedance mode, effectively protecting the hardware against transient overcurrents.

As the initial electromagnetic transient subsides and voltage fluctuations diminish, the system enters the electromechanical dynamic stage (*t* > 0^+^). During this phase, the virtual inductance stabilizes, and the adaptive control algorithm takes effect. Once the system satisfies the dynamic convergence criterion (i.e., Δ*ω*d*ω*/d*t* < 0) via state feedback, the algorithm drives a synchronized and gradual reduction of both *J* and *D*. This transition shifts the system from the preceding current-limiting state into a low-inertia mode. By actively reducing the inertia and damping support, the system mitigates the weak-grid-induced overdamped hysteresis, thereby enabling rapid active power regulation.

Ultimately, when the system completes the dynamic regulation process and returns to steady-state operation, the adaptive mechanism is deactivated. The virtual inductance *L*_vir_ returns to its nominal baseline value *L*_0_, while the virtual inertia *J* and damping coefficient *D* are restored to their rated design values *J*_0_ and *D*_0_, respectively. This coordinated multi-timescale approach utilizes the specific advantages of each control parameter, improving the coordination between dynamic response and steady-state performance under severe operating conditions.

## 4. Proposed Multi-Timescale Transient State Detection and Signal-Processing Framework

### 4.1. Measurement-Noise-Resilient Synergistic Sensing Strategy

To mitigate large frequency deviations and improve transient frequency support, the electromechanical parameters *J* and *D* must be dynamically adjusted based on the real-time grid state. Building upon the mathematical exponential synergistic mechanism proposed in our previous work [20], this section designs an enhanced adaptive framework specifically tailored for HESS hardware protection. In practical digital implementations, extracting the derivative of grid frequency is highly susceptible to hardware sensor noise and measurement inaccuracies, which can inadvertently trigger high-frequency control actions and increase battery current ripple or micro-cycling tendency. Thus, a robust first-order LPF is employed to obtain the filtered angular acceleration:

(13) $$G_{f}(s)=\frac{s}{T_{\omega }s+1}$$

where *T_ω_* is the filter time constant.

To balance noise attenuation with a rapid dynamic response, an adaptive buffering function for the virtual inertia *J* is formulated using exponential mapping:

(14) $$\left\{\begin{array}{ll} J=J_{0} & \left|\frac{d\omega }{dt}\right|\leq N\\ J=J_{0}+k_{1}{\left|\Delta \omega \frac{d\omega }{dt}\right|}^{\alpha }+k_{2}{\left|\Delta P\right|}^{\beta } & \left|\frac{d\omega }{dt}\right|>N\cap \Delta \omega \frac{d\omega }{dt}\geq 0\\ J=J_{0}-k_{1}{\left|\Delta \omega \frac{d\omega }{dt}\right|}^{\alpha }-k_{2}{\left|\Delta P\right|}^{\beta } & \left|\frac{d\omega }{dt}\right|>N\cap \Delta \omega \frac{d\omega }{dt}<0 \end{array}\right.$$

where *J*_0_ is the nominal inertia, *k*_1_ and *k*_2_ are the gain adjustment coefficients, *α* and *β* are the exponential adjustment coefficients, and *N* is the threshold of the angular acceleration.

To realize the intrinsic synergy between *J* and *D*, the optimal damping ratio of the system must be maintained during the dynamic tuning process. The relationship between *J* and *D* is obtained as

(15) $$J=\frac{\omega_{0}X}{4\xi^{2}U_{o}U_{g}}D^{2}$$

By substituting Equation (15) into Equation (14), the corresponding synergistic adaptive function for the damping coefficient *D* is derived as

(16) $$\left\{\begin{array}{ll} D=D_{0} & \left|\frac{d\omega }{dt}\right|\leq N\\ D=D_{0}\sqrt{1+\frac{1}{J_{0}}\left(k_{1}{\left|\Delta \omega \frac{d\omega }{dt}\right|}^{\alpha }+k_{2}{\left|\Delta P\right|}^{\beta }\right)} & \left|\frac{d\omega }{dt}\right|>N\cap \Delta \omega \frac{d\omega }{dt}\geq 0\\ D=D_{0}\sqrt{1-\frac{1}{J_{0}}\left(k_{1}{\left|\Delta \omega \frac{d\omega }{dt}\right|}^{\alpha }+k_{2}{\left|\Delta P\right|}^{\beta }\right)} & \left|\frac{d\omega }{dt}\right|>N\cap \Delta \omega \frac{d\omega }{dt}<0 \end{array}\right.$$

Through Equations (14) and (16), the coordinated strategy automatically increases *J* and *D* during the divergence phase (Δ*ω*d*ω*/d*t* ≥ 0) to suppress transient overshoot and actively decreases them during the convergence phase (Δ*ω*d*ω*/d*t* < 0) to mitigate the overdamped hysteresis commonly observed in weak grids.

### 4.2. Real-Time Voltage-Sag Detection and Active Electromagnetic Shielding

In weak grid environments, abrupt changes in grid impedance or severe short-circuit faults typically induce transient voltage sags at the point of common coupling (PCC). Traditional fixed virtual impedance control presents an inherent trade-off: it cannot simultaneously provide accurate steady-state voltage regulation and robust transient overcurrent suppression. To address this limitation, an exponential adaptive virtual inductance strategy based on PCC voltage deviation is proposed.

By utilizing the depth of the voltage sag as the core dynamic variable, the initial adaptive function is formulated as

(17) $$L_{\mathrm{adapt}}=k_{\mathrm{vir}}(1-e^{-\lambda \left|U_{\mathrm{ref}}-U_{\mathrm{pcc}}\right|})$$

where *k*_vir_ is the adaptive gain, λ is the sensitivity coefficient, *U*_ref_ is the reference voltage amplitude, and *U*_pcc_ is the actual measured PCC voltage amplitude. This exponential formulation allows the virtual inductance command *L*_adapt_ to increase nonlinearly as the voltage drop becomes larger, thereby providing additional damping support for inrush-current suppression.

To prevent undesirable fluctuations of the virtual inductance *L*_vir_ triggered by high-frequency voltage noise or instantaneous measurement jumps, a first-order LPF is cascaded at the output of the adaptive function. The filtered output is then superimposed on a steady-state baseline inductance *L*_0_, yielding the final implemented virtual inductance:

(18) $$L_{\mathrm{vir}}(s)=\frac{1}{T_{f}s+1}L_{\mathrm{adapt}}+L_{0}$$

where *T_f_* is the filter time constant.

The integration of the LPF and the baseline inductance plays a pivotal role in optimizing the dynamic transition of the system. At the instant of a grid fault, the abrupt drop in PCC voltage causes a step change in the calculated *L*_adapt_. The LPF helps the effective *L*_vir_ follow a smooth rising trajectory, which reduces the possibility of secondary electromechanical oscillations induced by abrupt impedance variations.

Simultaneously, this provides a gradually increasing damping force, assisting the system in transitioning smoothly from a severe transient state back to a stable steady state. Furthermore, the baseline inductance *L*_0_ provides a minimum electrical stiffness and power-decoupling capability during normal steady-state operation, preventing the grid-forming inverter from becoming vulnerable to minor disturbances when *L*_adapt_ returns to zero.

### 4.3. Sustainable Parameter Optimization and Physical Constraints

#### 4.3.1. Hardware Safety Boundaries

An unrestricted increase in *J* may require a large transient energy exchange, which could increase battery stress and raise the risk of DC-link voltage deviation. Therefore, the sensing framework should bound the adaptive parameters to limit the transient energy exchange on the DC side. Thus, *J* must be strictly constrained by the maximum transient power capability of the inverter (*P*_max_):

(19) $$J\leq J_{\max}=\frac{P_{\max}-P_{\mathrm{ref}}}{\omega_{0}{\left|\frac{d\omega }{dt}\right|}_{\max}}$$

Similarly, the damping coefficient *D* requires an upper bound. Although a larger *D* improves the attenuation of transient oscillations, an excessive damping value introduces steady-state tracking errors, thereby compromising overall control accuracy. According to the fundamental swing equation, under stable steady-state conditions (where d*ω*/d*t* = 0), the upper limit for the damping coefficient is dictated by the maximum mechanical torque (*T*_mmax_) and the maximum allowable angular frequency deviation (*ω*_max_):

(20) $$0<D<\frac{T_{\mathrm{mmax}}-T_{e}}{\omega_{\max}-\omega_{0}}$$

#### 4.3.2. Sensor Noise Immunity Threshold and Filter Sensitivity Tuning

The threshold N essentially functions as a noise-rejection dead-band for the sensing framework. In practical PV-storage plants, frequency measurement units or PLLs introduce high-frequency sensor noise (*ω*_noise_). If the adaptive algorithm is overly sensitive, this noise can inadvertently trigger the exponential functions, increasing high-frequency battery current ripple and possibly leading to a micro-cycling tendency. To maximize economic sustainability, the dead-band *N* must satisfy the background noise constraint:

(21) $$\max\left|{\left(\frac{d\omega }{dt}\right)}_{\mathrm{noise}}\right|<N<\left|{\left(\frac{d\omega }{dt}\right)}_{\mathrm{limit}}\right|$$

Specifically, the lower bound of the dead-band must exceed the maximum residual rate of change of the measurement noise after passing through the LPF. Conversely, the upper bound should remain below the activation level associated with real electromechanical transients, so that actual load or frequency disturbances can still be detected without excessive delay. In this study, *N* = 2.0 rad/s^2^ is selected as the dead-band threshold for the filtered angular acceleration signal. The unit of *N* is consistent with the filtered frequency derivative d*ω_f_*/d*t*. Therefore, *N* is not an arbitrary dimensionless coefficient but a physical angular-acceleration threshold. The selection of *N* follows two requirements: it should be larger than the residual derivative-noise level after LPF processing to avoid repeated noise-induced activation of the adaptive *J*-*D* loop, and it should remain lower than the activation level of real electromechanical transients to preserve dynamic responsiveness. The sensitivity of *N* = 1, 2, 3 rad/s^2^ is further evaluated in Section 5.4, where the trade-off between false-trigger suppression and activation delay is quantified.

The exponential coefficients (*α*, *β*) are responsible for reshaping the dynamic electromechanical buffering trajectory. To achieve a smooth initial activation combined with robust transient suppression during sudden faults, the acceleration-driven exponent is designed as *α* = 1.5 (where *α* > 1). This non-linearity ensures a moderate inertia response during small load variations while providing a stronger response during large grid disturbances. Conversely, the deviation-driven exponent is set to *β* = 0.8 (where *β* < 1) to provide a sustained damping effect during the final steady-state convergence, thereby helping reduce residual hysteresis oscillations.

Furthermore, the scaling gains (*k*_1_ = 1.8, *k*_2_ = 1.6) are selected by considering the maximum allowable transient energy exchange constraint of the DC bus:

(22) $$\int_{t_{0}}^{t_{\mathrm{clear}}}\Delta P_{\mathrm{surge}}dt\leq E_{\mathrm{bat}\_\mathrm{margin}}$$

where *t*_0_ and *t*_clear_ denote the onset and clearance instants of the grid fault, respectively; Δ*P*_surge_ represents the instantaneous active power surge induced by the grid shock; and *E*_bat_margin_ indicates the maximum allowable transient energy margin of the battery system. This integral constraint helps limit the transient power surge associated with the dynamic *J* and *D* parameters, thereby reducing the risk of DC/DC converter over-current protection activation and DC-link voltage collapse during fault ride-through.

The parameter tuning for the adaptive virtual inductance balances active/reactive power decoupling with voltage regulation. The nominal steady-state baseline inductance is set to *L*_0_ = 3*R*_g_/*ω*_0_ to satisfy the fundamental *X*/*R* inductive decoupling prerequisite under weak grid conditions.

The frequency-derivative LPF time constant *T_ω_* determines the filtering strength in the adaptive *J*-*D* sensing path. A smaller *T_ω_* improves response speed but allows more derivative noise to pass through, while a larger *T_ω_* improves noise attenuation but increases activation delay. In the simulations, *T_ω_* = 10 ms is selected as the nominal value; the sensitivity of *T_ω_* = 5, 10, 20 ms is evaluated in Section 5.4. Therefore, *T_ω_* is selected according to the compromise between derivative ripple suppression and transient detection delay.

Additionally, the filter time constant *T_f_* dictates the response speed of the transient impedance reshaping. An overly large value introduces hazardous response latency during short-circuits, while a value too small compromises the filtering efficacy. Thus, *T_f_* is set to 0.02 s (corresponding to a cutoff frequency of approximately 8 Hz), which attenuates high-frequency noise while maintaining the ability to track abrupt voltage sags. To prevent false triggering, the adaptive gain *k*_vir_ and sensitivity *λ* are configured such that the exponential function is sharply activated only when the voltage sag depth exceeds 10%. Through simulation testing, the adopted parameters are selected as *λ* = 0.05 and *k*_vir_ = 0.005.

To clarify the practical implementation of the proposed adaptive sensing strategy, the real-time update logic is summarized as follows. At each control sampling instant, the PCC voltage, output current, active/reactive power, and angular frequency are measured. The raw frequency derivative is first filtered by the LPF with time constant *T_ω_*. If the filtered angular acceleration remains within the dead-band [−*N*,*N*], the adaptive inertia and damping loop remains inactive, and the nominal values *J*_0_ and *D*_0_ are retained. Once the threshold is exceeded, the transient dynamic state is identified using the sign of Δ*ω*d*ω*/d*t*, and the virtual inertia *J* and damping coefficient *D* are updated according to the exponential adaptive functions. In parallel, the PCC voltage deviation ∣*U*_ref_ − *U*_pcc_∣ is monitored. When a significant voltage sag is detected, the adaptive virtual inductance command is generated by the voltage-deviation function, filtered by the LPF with time constant *T_f_*, and superimposed on the baseline inductance *L*_0_. The resulting *L*_vir_ is then used in the virtual-impedance loop for transient current suppression. During implementation, all adaptive parameters are bounded by their admissible ranges, namely *J*_min_ ≤ *J* ≤ *J*_max_, *D*_min_ ≤ *D* ≤ *D*_max_, and *L*_0_ ≤ *L*_vir_ ≤ *L*_max_. These bounds are selected according to the converter current limit, DC-link energy margin, and acceptable PCC voltage drop.

### 4.4. Reduced-Order Stability Assessment via Frozen-Coefficient Analysis

Since the virtual inertia *J* and damping coefficient *D* are adaptively updated according to the measured transient state, the active-power/frequency loop of the VSG becomes a time-varying nonlinear system. A full-order stability proof of the complete converter-HESS system would require the simultaneous consideration of the inner current and voltage loops, current saturation, digital delay, voltage-loop interaction, and the dynamics of the battery and supercapacitor converters. Such a complete proof is beyond the scope of the reduced-order model considered in this subsection. Therefore, the following analysis is limited to a frozen-coefficient small-signal assessment of the outer active-power/frequency loop.

Within one digital sampling interval *t*_k_, the adaptive parameters *J*(*t*_k_) and *D*(*t*_k_) are assumed to be quasi-static. Under this frozen-coefficient assumption, the equivalent characteristic equation of the reduced-order VSG active-power/frequency loop can be expressed as

(23) $$s^{2}+\frac{D(t_{k})}{J(t_{k})}s+\frac{K_{s}}{J(t_{k})\omega_{0}}=0$$

where *K*_s_ is the equivalent synchronizing power coefficient. Under the tested weak-grid operating conditions, *K*_s_ remains positive around the selected operating point. Therefore, the dominant poles of the reduced-order active-power/frequency loop are given by

(24) $$s_{1,2}=-\frac{D(t_{k})}{2J(t_{k})}\pm j\sqrt{\frac{K_{s}}{J(t_{k})\omega_{0}}-{\left(\frac{D(t_{k})}{2J(t_{k})}\right)}^{2}}$$

According to the parameter constraints introduced in Section 4.3, the adaptive parameters are bounded as *J*(*t*_k_) ≥ *J*_min_ > 0 and *D*(*t*_k_) ≥ *D*_min_ > 0. Therefore, for the reduced-order frozen-coefficient model, the real part of the dominant poles is

(25) Re(s1,2)=−D(tk)2J(tk)<0

**Figure 8:**
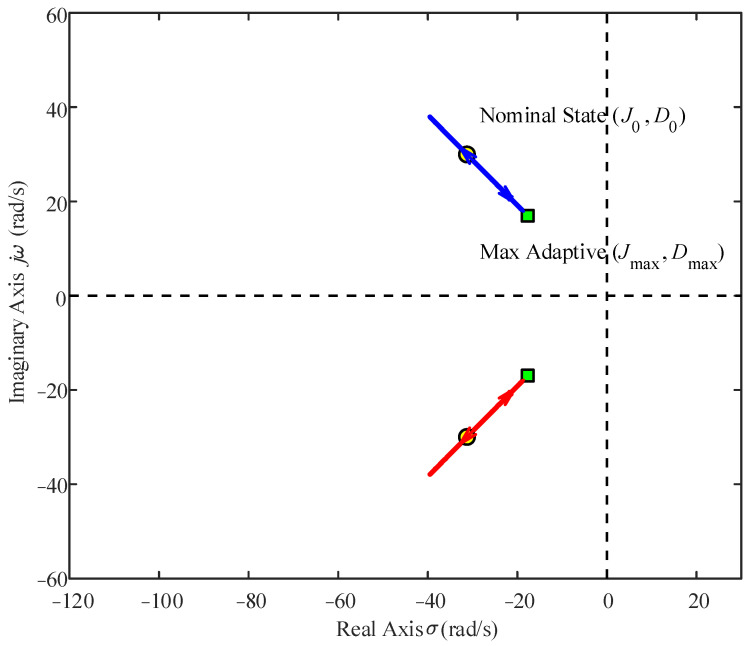
Reduced-order frozen-coefficient pole-location assessment of the VSG active-power/frequency loop under bounded *J*(*t*_k_) and *D*(*t*_k_). The yellow circles denote the dominant pole locations at the nominal operating state (*J*_0_, *D*_0_); the green squares denote the dominant pole locations at the maximum adaptive state (*J*_max_, *D*_max_). The blue and red arrows indicate the movement trends of the upper and lower conjugate poles, respectively, as *J*(*t*_k_) and *D*(*t*_k_) vary within the bounded adaptive range.

This indicates that the local active-power/frequency dynamics remain damped under the frozen *J*(*t*_k_) and *D*(*t*_k_) assumptions. The corresponding pole distribution under the considered bounded parameter range is shown in Figure 8.

To further illustrate the local damping tendency of the reduced-order active-power/frequency loop, the dominant pole locations under different frozen *J*(*t*_k_) and *D*(*t*_k_) values are shown in Figure 8. It can be observed that the poles remain in the left-half plane within the considered bounded parameter range, indicating that the outer electromechanical loop preserves local damping under the quasi-static *J*(*t*_k_), *D*(*t*_k_), and positive-*K*_s_ assumptions. It should be noted that Figure 8 is only used as a reduced-order pole-location assessment of the active-power/frequency loop rather than as a global transient stability proof of the complete converter-HESS system, since inner current/voltage loops, saturation, digital delay, voltage-loop interaction, and HESS converter dynamics are not included in this reduced-order analysis.

## 5. Simulation Analysis and Discussion

Based on the preceding theoretical analysis, a grid-connected PV-hybrid storage VSG simulation platform is developed in MATLAB R2024b/Simulink to evaluate the proposed multi-timescale transient-state sensing and control strategy. The simulated system consists of a PV generation unit, a battery-supercapacitor HESS, a grid-forming inverter, an LCL filter, and an equivalent weak-grid interface. The main simulation parameters are listed in Table 1.

To clearly assess the contribution of each functional module, the simulation analysis is organized into four groups. First, source-side irradiance step disturbances are applied to verify the frequency-domain HESS power-decoupling mechanism and its effect on DC-link and battery current stress. Second, different grid-strength conditions are considered to evaluate the adaptive virtual inertia and damping strategy for active-power and frequency regulation in weak grids. Third, a three-phase voltage-sag fault is introduced to examine the voltage-deviation-driven adaptive virtual inductance and its transient inrush-current suppression capability. Fourth, measurement-noise perturbation and parameter-sensitivity tests are performed to verify the robustness of the filtered frequency-derivative sensing and dead-band design.

For comparison, the proposed strategy is evaluated against representative baseline and ablation cases under the same electrical parameters and disturbance conditions. The fixed-parameter VSG keeps *J* = *J*_0_, *D* = *D*_0_, and *L*_vir_ = *L*_0_ and disables the proposed transient-state sensing loops. The adaptive *J*,*D*-only case enables the frequency-derivative-based adaptive inertia and damping regulation while keeping *L*_vir_ = *L*_0_. The adaptive *L*_vir_-only case enables voltage-deviation-driven virtual inductance regulation while keeping *J* = *J*_0_ and *D* = *D*_0_. The proposed strategy enables both adaptive *J*, *D* and adaptive *L*_vir_, together with the HESS power-decoupling and noise-resilient sensing loops. This definition is used consistently in the comparative simulations of Section 5.2, Section 5.3, Section 5.4 and Section 5.5.

To improve the reproducibility of the comparative simulations, the supplementary simulation settings and the definitions of the comparison cases are summarized in Table 2. Unless otherwise specified, the same electrical parameters listed in Table 1 are used for all cases.

It should be noted that the 10 kW value denotes the nominal pre-disturbance active-power operating point used for the grid-forming power-control tests. The large current and power values observed during voltage-sag simulations are instantaneous transient peaks caused by the deliberately imposed fault condition, rather than continuous operating ratings. These peak values correspond to short-duration fault-stress responses in the simulation model and are used only to compare the current-limiting capability of different control strategies under the same voltage-sag scenario. They should not be interpreted as the continuous current rating of the converter. The electrical base values, converter rating, sampling settings, and comparison-case definitions are listed in Table 1 and Table 2 to clarify the reproducibility of the simulations.

### 5.1. HESS Power-Decoupling Performance Under Step Irradiance Disturbances

**Figure 9:**
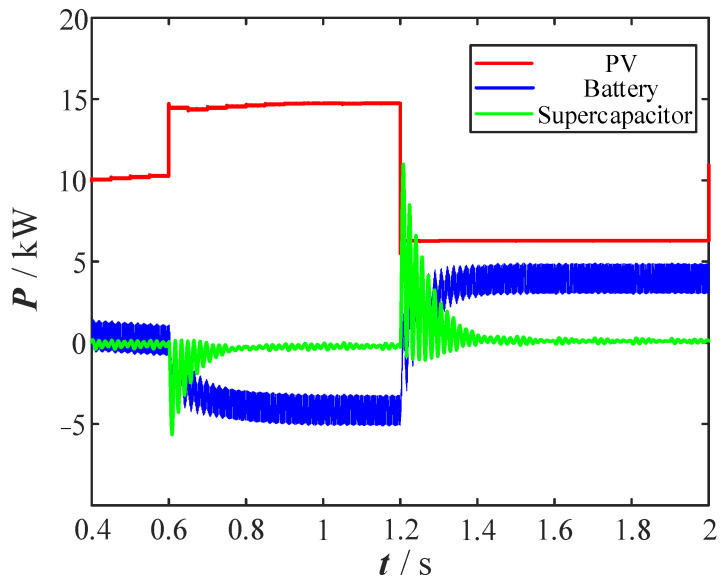
Dynamic power allocation among the PV array and hybrid energy storage units under step changes in solar irradiance.

**Figure 10:**
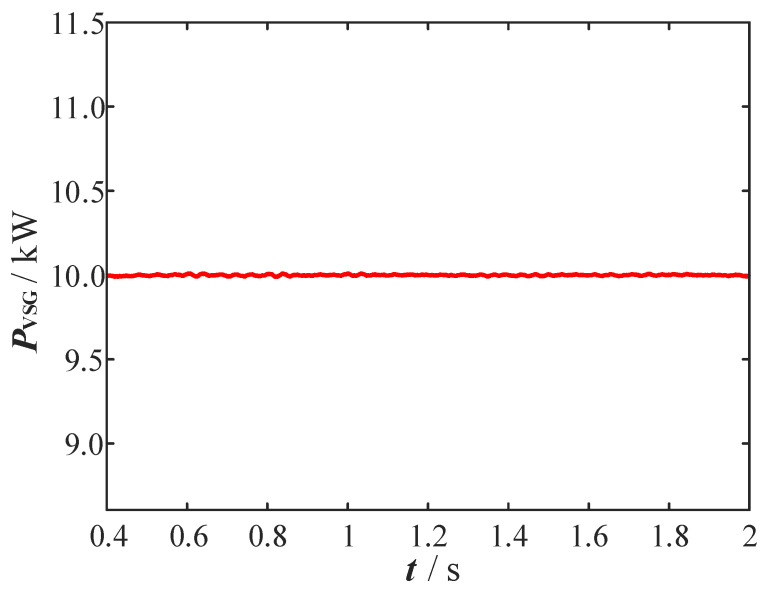
Output active power response of the grid-connected VSG under source-side disturbances.

**Figure 11:**
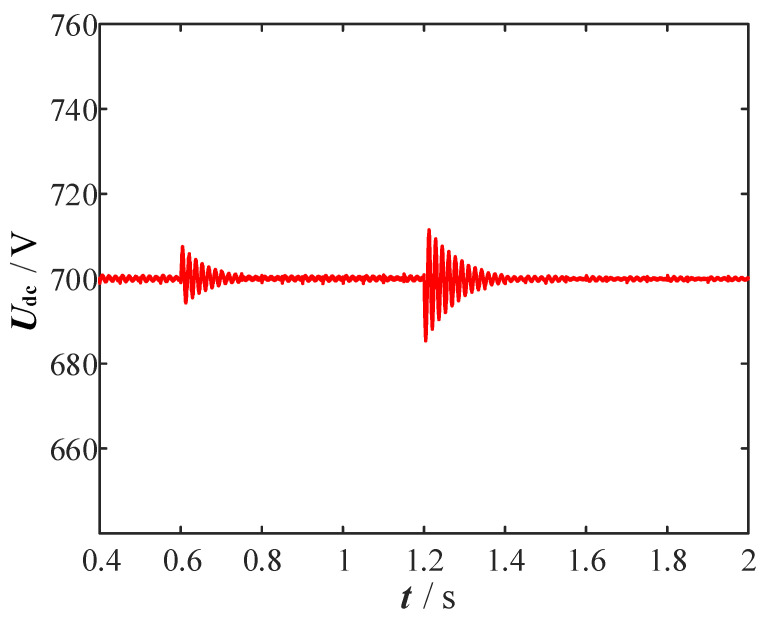
Dynamic response and stabilization of the DC bus voltage.

**Figure 12:**
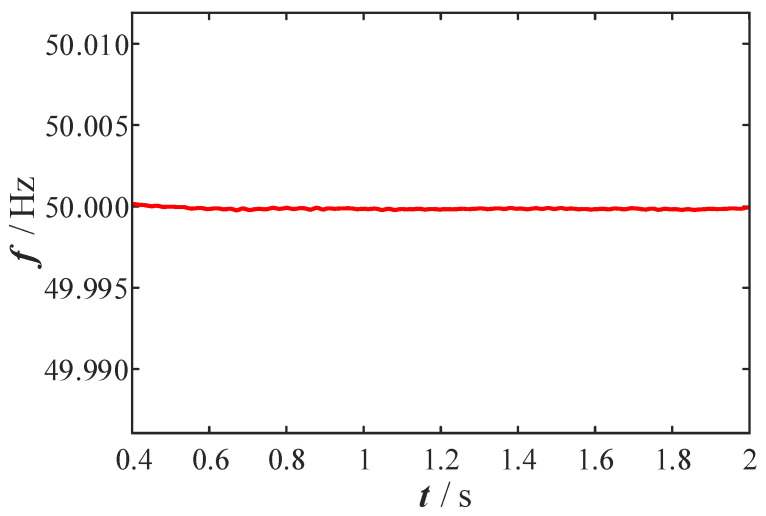
System AC frequency response during the irradiance step variations.

To verify the energy management and coordinated control capability of the PV-hybrid storage system under severe source-side input power fluctuations, step changes in solar irradiance are simulated. The initial irradiance is set to 1000 W/m^2^, the ambient temperature is maintained at 25 °C, and the VSG active-power reference is kept constant at 10 kW. At *t* = 0.6 s, the irradiance increases to 1400 W/m^2^; subsequently, at *t* = 1.2 s, the irradiance decreases to 600 W/m^2^. The corresponding dynamic responses, including the power distribution among the PV array and HESS units, the grid-forming VSG active-power output, the DC-link voltage, and the system frequency, are shown in Figure 9, Figure 10, Figure 11 and Figure 12.

In addition to waveform-based power allocation, this test is further used to evaluate battery-current stress indicators under the proposed HESS frequency-domain decoupling mechanism.

Figure 9 illustrates the dynamic power allocation among the PV array, battery, and supercapacitor under the two irradiance step disturbances. Before *t* = 0.6 s, the PV output power is close to the 10 kW grid-forming power reference, and the HESS remains near its steady operating condition. When the irradiance increases from 1000 W/m^2^ to 1400 W/m^2^ at t = 0.6 s, the PV output power rises rapidly from approximately 10.2 kW to 14.8 kW, producing a surplus power of about 4.8 kW on the DC side. At this moment, the supercapacitor responds first and absorbs the fast-changing component of the surplus power, with its charging power reaching approximately 5 kW. This response is consistent with the high-power-density characteristic of the supercapacitor and the frequency-domain decoupling principle of the HESS controller. After the initial transient, the battery gradually participates in the power balancing process and absorbs the slower-varying energy component. Therefore, the battery is not required to directly follow the steep PV power step, which helps reduce high-frequency current stress imposition on the battery.

When the irradiance decreases to 600 W/m^2^ at *t* = 1.2 s, the PV output power drops to approximately 6 kW, resulting in a power deficit relative to the 10 kW grid-forming output requirement. In this case, the supercapacitor changes from charging to discharging and provides rapid compensation for the transient power shortage. Its discharge power reaches approximately 11 kW during the initial compensation interval. After the fast transient component is compensated by the supercapacitor, the battery gradually takes over the lower-frequency energy demand and supplies about 4 kW to support the DC-side power balance. This two-stage response indicates that the HESS controller assigns high-frequency power variations to the supercapacitor and low-frequency energy variations to the battery, thereby realizing the intended frequency-domain power decoupling.

Figure 10 shows the active-power response of the grid-forming VSG under the same source-side irradiance disturbances. Although the PV output power changes sharply at *t* = 0.6 s and *t* = 1.2 s, the VSG output active power remains regulated around the 10 kW reference. This result indicates that the DC-side HESS absorbs or supplies the mismatch between PV generation and grid-forming output power before the disturbance propagates significantly to the AC side. In other words, the HESS does not simply smooth the PV power curve, it also provides a buffer between the stochastic source-side input and the regulated grid-forming power output. The power exchange among the PV array, battery, supercapacitor, and inverter remains consistent with the system power-balance relationship described in Equation (1).

Figure 11 presents the DC-link voltage response during the irradiance step variations. Since the DC bus is the coupling point between the PV source, the HESS, and the grid-forming inverter, its voltage response directly reflects the internal energy balance of the PV-storage system. At *t* = 0.6 s, the sudden increase in PV power causes a short voltage rise. The maximum voltage overshoot is approximately 8 V, corresponding to about 1.1% of the nominal 700 V DC-link voltage. After the HESS absorbs the surplus transient energy, the DC-link voltage returns to the 700 V reference within approximately 0.1 s. Similarly, when the irradiance decreases at *t* = 1.2 s, the DC-link voltage exhibits only a limited transient deviation and then quickly converges back to the reference value. These results show that the HESS controller provides a fast DC-side energy-buffering path and prevents large DC-link voltage excursions during severe irradiance disturbances.

Figure 12 shows the AC-side frequency response during the same test. Since the VSG active-power reference is maintained at 10 kW and the source-side power disturbance is mainly compensated by the HESS, the system frequency remains close to the rated 50 Hz throughout the irradiance variation process. Only negligible transient fluctuations are observed during the two irradiance steps. This result further confirms that the proposed HESS power-decoupling mechanism reduces the direct propagation of PV-side power fluctuations to the AC-side frequency-control loop. Therefore, the HESS module mainly functions as a fast DC-side energy buffer, while the VSG maintains the grid-forming active-power and frequency response.

Overall, Figure 9, Figure 10, Figure 11 and Figure 12 demonstrate that the proposed HESS-based frequency-domain power-decoupling strategy can coordinate the complementary characteristics of the battery and supercapacitor under large irradiance variations. The supercapacitor provides fast transient power compensation, while the battery handles slower energy balancing. As a result, the grid-forming inverter maintains a stable active-power output, the DC-link voltage remains within a small deviation range, and the AC frequency is kept close to its nominal value. These results verify the effectiveness of the HESS module in reducing the influence of source-side PV fluctuations on both the DC bus and the AC-side grid-forming response.

To further quantify the contribution of the HESS power-decoupling mechanism to battery-stress reduction, several current-based indicators are calculated under the same irradiance-step test. The comparison baseline is a single-battery compensation case, in which the complete PV–VSG power mismatch is assumed to be handled by the battery without the supercapacitor high-frequency branch. The proposed case uses the LPF-based HESS power decoupling shown in Figure 3. The calculation interval is selected as 0.2–2.0 s to exclude the initial numerical start-up transient and focus on the irradiance-step response. It should be emphasized that these indicators are not intended to replace a full electrochemical aging model. Instead, they provide measurable evidence of battery-current stress reduction, which is directly related to current ripple, charge/discharge reversal, and micro-cycling tendency.

The battery RMS current is calculated as

(26) $$I_{\mathrm{bat},\mathrm{RMS}}=\sqrt{\frac{1}{T}\int_{t_{0}}^{t_{1}}i_{\mathrm{bat}}^{2}(t)dt}$$

The high-frequency battery-current component is obtained from

(27) $$i_{\mathrm{bat},\mathrm{HF}}(t)=i_{\mathrm{bat}}(t)-i_{\mathrm{bat},\mathrm{LF}}(t)$$

where *i*_bat,LF_(*t*) is obtained using a moving-average low-pass estimate consistent with the HESS decoupling principle. The corresponding high-frequency RMS current is calculated as

(28) $$I_{\mathrm{bat},\mathrm{HF},\mathrm{RMS}}=\sqrt{\frac{1}{T}\int_{t_{0}}^{t_{1}}i_{\mathrm{bat},\mathrm{HF}}^{2}(t)dt}$$

The battery charge throughput during the tested interval is defined as

(29) $$Q_{\mathrm{bat},\mathrm{thr}}=\frac{1}{3600}\int_{t_{0}}^{t_{1}}|i_{\mathrm{bat}}(t)|dt$$

The calculated battery-current stress indicators are summarized in Table 3.

As shown in the current-stress indicators, the proposed HESS decoupling strategy reduces the battery RMS current from 7.648 A to 6.738 A, corresponding to an 11.9% reduction. More importantly, the high-frequency battery-current RMS decreases from 2.393 A to 1.176 A, corresponding to a 50.8% reduction. The battery peak current is also reduced from 25.301 A to 10.240 A, indicating that the supercapacitor branch absorbs a substantial portion of the fast transient power component during irradiance steps. In addition, the battery charge throughput over the tested interval decreases from 3.38 mAh to 3.00 mAh. These results provide quantitative current-stress evidence that the proposed HESS mechanism reduces the direct exposure of the battery to high-frequency transient power. Therefore, the proposed strategy reduces measurable battery-current stress and may help mitigate battery micro-cycling tendency under the tested irradiance-step condition. However, a complete battery lifetime prediction would require an electrochemical aging model, thermal coupling, and long-term cycling tests, which are beyond the scope of this work.

### 5.2. Active-Power, Frequency, and Current Responses Under Varying Grid Strengths

**Figure 13:**
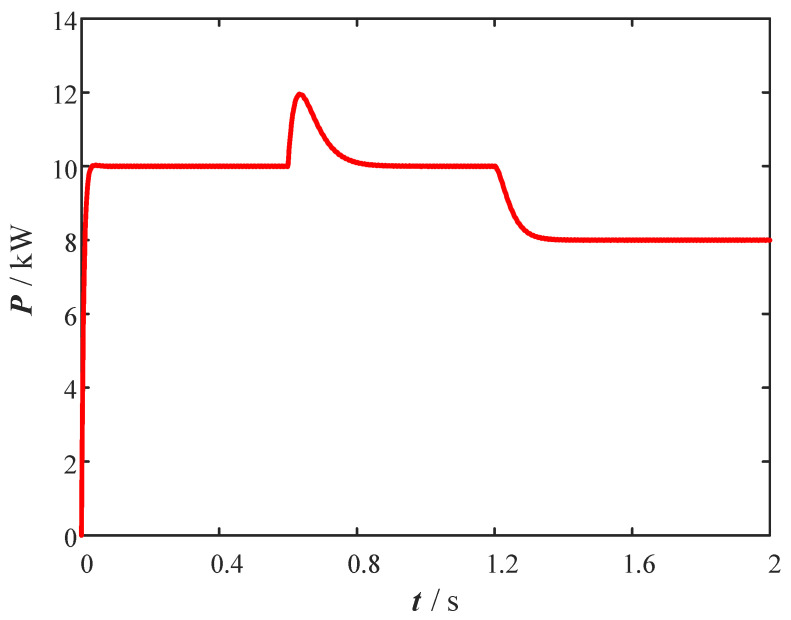
Active power response under the strong grid condition (SCR = 20).

**Figure 14:**
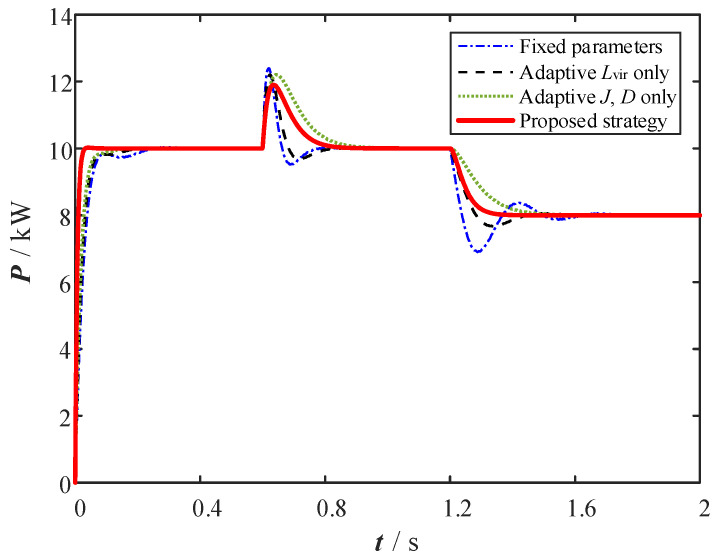
Comparison of active power responses under the extremely weak grid condition (SCR = 2.5).

**Figure 15:**
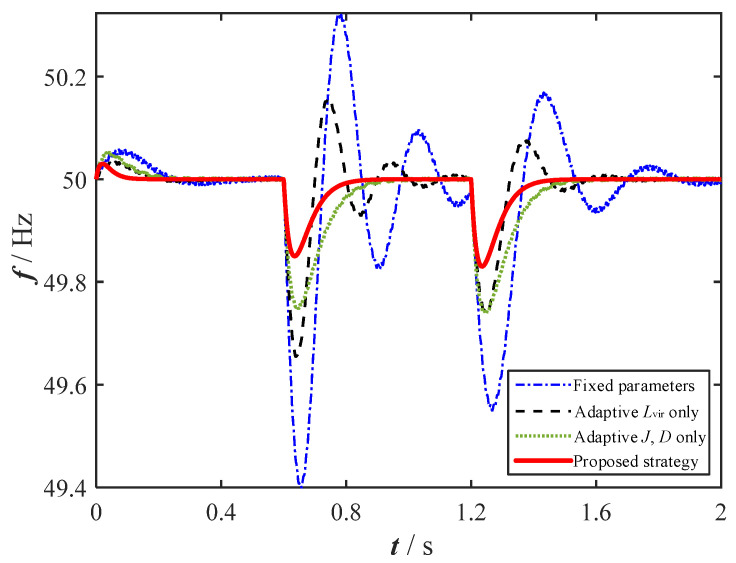
Comparison of system frequency responses under the weak grid condition (SCR = 2.5).

**Figure 16:**
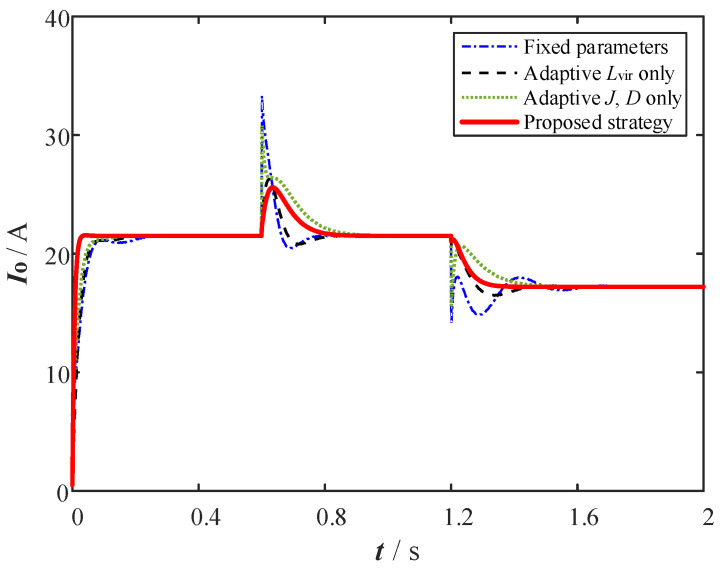
Comparison of output current waveforms under the weak grid condition (SCR = 2.5).

To evaluate the adaptability of the proposed strategy under different grid-strength conditions, two scenarios are considered: a strong grid with SCR = 20 and an extremely weak grid with SCR = 2.5. The system initially operates at 10 kW. At *t* = 0.6 s, a 2 kW local load is connected, and at *t* = 1.2 s, the active-power reference is reduced from 10 kW to 8 kW. The corresponding active-power, frequency, and output-current responses are shown in Figure 13, Figure 14, Figure 15 and Figure 16.

Figure 13 shows the active-power response under the strong grid condition. Because the grid impedance is low and the power-transfer margin is sufficient, all tested strategies maintain acceptable tracking performance after the load disturbance and reference change. The active power converges to the corresponding operating point without obvious long-lasting oscillations, indicating that the proposed adaptive strategy does not introduce noticeable adverse effects under strong-grid operation.

Figure 14 compares the active-power responses under the extremely weak grid condition. When SCR decreases to 2.5, the fixed-parameter VSG exhibits a clear transient overshoot after the load disturbance and a dynamic undershoot after the reference reduction. Specifically, the fixed-parameter case shows about 24% active-power overshoot at *t* = 0.6 s and about 31% undershoot at *t* = 1.2 s. These results indicate that fixed inertia and damping are not sufficient when the grid impedance significantly restricts transient power transfer. In contrast, the proposed strategy suppresses the overshoot to below 19% and accelerates the active-power recovery by more than 50%, showing improved adaptability under weak grid conditions.

Figure 15 presents the frequency responses under the weak grid condition. The fixed-parameter VSG produces the largest frequency deviation, approximately 0.6 Hz, and is followed by secondary oscillations. The single-function adaptive strategies reduce the deviation to about 0.35 Hz and 0.25 Hz. With the proposed coordinated strategy, the maximum frequency deviation is further reduced to approximately 0.15 Hz, corresponding to about a 75% reduction compared with the fixed-parameter VSG. This result verifies that coordinated inertia–damping adaptation can improve frequency support in high-impedance grids.

Figure 16 compares the output-current responses. Under the fixed-parameter VSG, the load disturbance causes a current surge exceeding 1.5 p.u., while the adaptive inertia–damping-only strategy still results in a peak current of about 1.4 p.u. By contrast, the proposed strategy limits the current envelope to approximately 1.15 p.u. This improvement is mainly attributed to the adaptive virtual inductance, which increases the equivalent output impedance during the transient interval and reduces converter current stress.

Overall, Figure 13, Figure 14, Figure 15 and Figure 16 show that the proposed multi-timescale strategy improves active-power convergence, frequency stability, and transient current suppression under weak-grid conditions. Compared with fixed-parameter VSG control, the proposed method better coordinates electromechanical-timescale inertia–damping adaptation and electromagnetic-timescale virtual impedance regulation, thereby enhancing the dynamic resilience of the PV-storage system in high-impedance grids.

### 5.3. Voltage-Sag Sensing and Transient Inrush-Current Suppression

**Figure 17:**
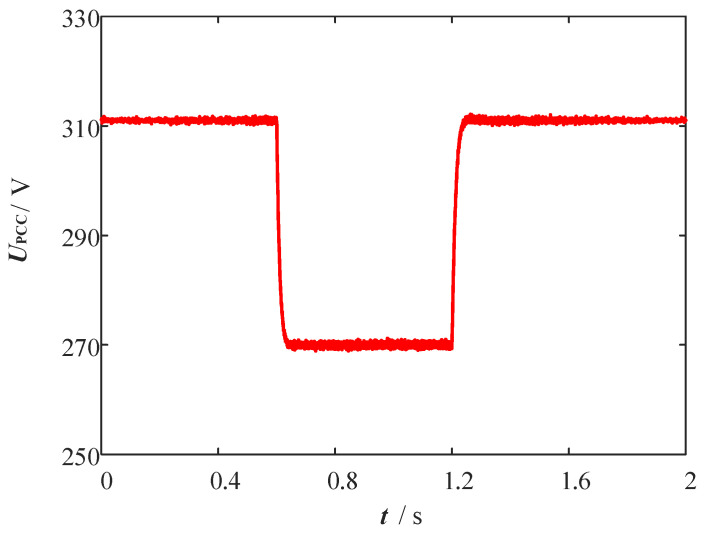
Transient variation of the voltage at the Point of Common Coupling (PCC).

**Figure 18:**
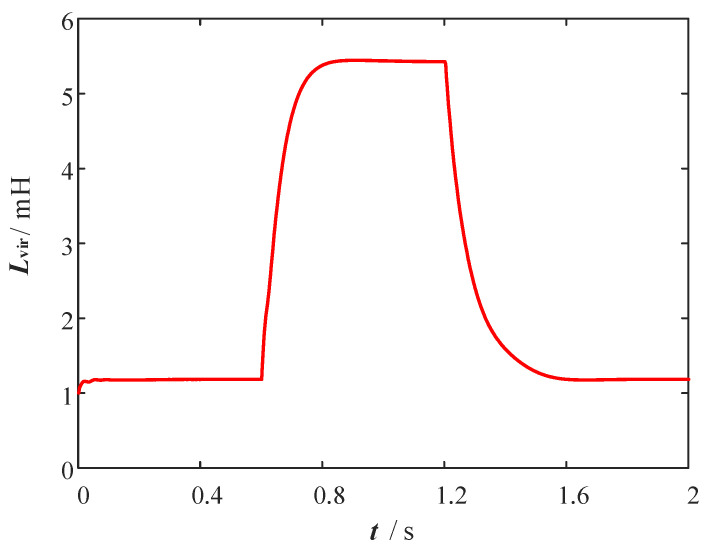
Dynamic evolution of the adaptive virtual inductance *L*_vir_ during the fault.

**Figure 19:**
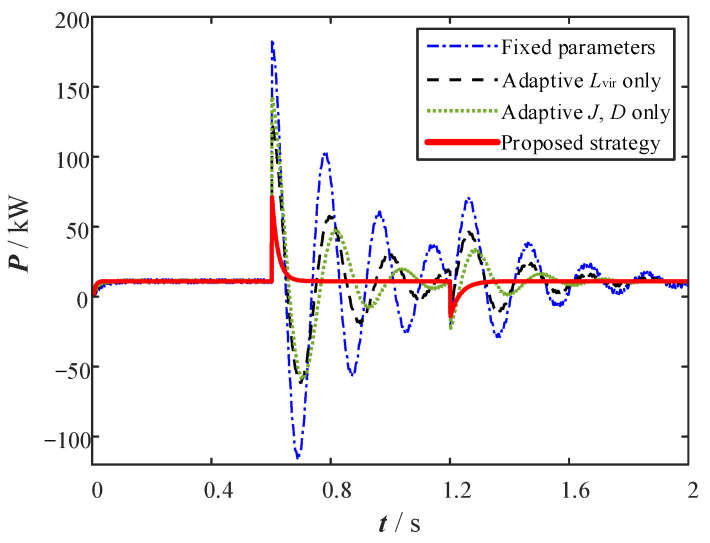
Comparison of active power responses under severe grid voltage sag.

**Figure 20:**
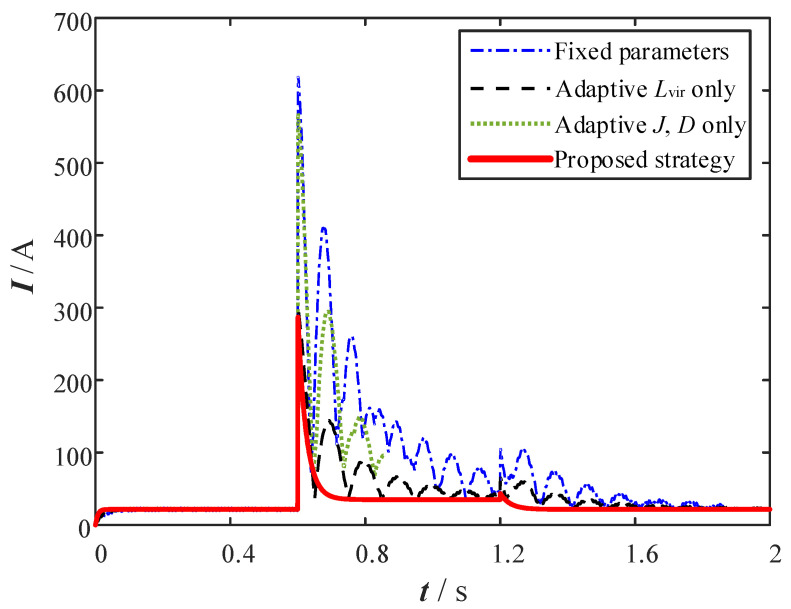
Comparison of output current waveforms and transient inrush suppression.

To verify the fault ride-through capability and transient current-limiting performance of the proposed strategy, a three-phase symmetrical voltage sag is applied at *t* = 0.6 s and cleared at *t* = 1.2 s. The PCC voltage, adaptive virtual inductance, active-power response, and output-current response under different control strategies are shown in Figure 17, Figure 18, Figure 19 and Figure 20.

Figure 17 shows the PCC voltage response during the voltage-sag event. At *t* = 0.6 s, the PCC voltage drops from approximately 311 V to about 270 V, corresponding to a voltage-sag depth of 13.2%. After the fault is cleared at *t* = 1.2 s, the PCC voltage recovers toward its pre-fault value. This voltage deviation provides the triggering signal for the proposed voltage-sag sensing loop.

Figure 18 presents the dynamic evolution of the adaptive virtual inductance. When the PCC voltage sag is detected, the proposed algorithm increases *L*_vir_ from its steady-state baseline of 1.2 mH to approximately 5.4 mH. This increase raises the equivalent output impedance of the converter during the fault interval. Meanwhile, the low-pass filter in the virtual-inductance path smooths the transition of *L*_vir_, avoiding an abrupt impedance jump that could introduce additional oscillations.

Figure 19 compares the active-power responses under different control strategies during the voltage sag. The fixed-parameter VSG exhibits a large transient power disturbance and a long recovery process after the fault occurs and clears. The adaptive *J*, *D* only strategy improves the electromechanical response, but it cannot directly limit the electromagnetic-timescale interaction between the converter and the faulted grid. The adaptive *L*_vir_ only strategy improves current limiting, but the active-power response still contains noticeable oscillations. In contrast, the proposed coordinated strategy reduces the transient power fluctuation and provides a smoother recovery after the fault is cleared. This result indicates that current-limiting impedance adaptation and inertia–damping coordination are both necessary for fault ride-through under weak-grid conditions.

The 10 kW value denotes the nominal pre-disturbance active-power operating point of the PV-storage VSG. In contrast, the large instantaneous power and current peaks observed during the voltage-sag test are short-duration transient fault-stress quantities caused by the sudden PCC voltage drop and the resulting electromagnetic energy exchange among the filter, grid impedance, and converter output. These transient peaks are used only for relative comparison among different control strategies under the same fault condition and should not be interpreted as continuous operating ratings of the converter or storage system.

Figure 20 further compares the output-current responses. These current values represent short-duration instantaneous peak currents during the voltage-sag stress test and are used for relative comparison among the tested strategies. At the onset of the voltage sag, the fixed-parameter VSG produces a large inrush-current peak of about 620 A. The adaptive *J*, *D* only strategy reduces the peak only slightly to approximately 580 A, showing that electromechanical parameter tuning alone is insufficient for converter overcurrent protection. The adaptive *L*_vir_ only strategy reduces the initial current peak to about 310 A, but the current envelope still contains noticeable fluctuations due to the remaining power oscillations. By contrast, the proposed coordinated strategy limits the maximum inrush current to approximately 290 A, corresponding to a 53.2% reduction compared with the fixed-parameter VSG. The current waveform also becomes smoother after the initial impact, indicating improved coordination between transient current suppression and dynamic power regulation.

Overall, Figure 17, Figure 18, Figure 19 and Figure 20 demonstrate that the proposed voltage-sag sensing strategy can detect the PCC voltage deviation and increase the virtual inductance during the fault interval. Compared with fixed-parameter and single-function adaptive strategies, the proposed multi-timescale strategy provides a better balance between voltage-sag ride-through, inrush-current suppression, and active-power recovery. These results support the effectiveness of combining electromagnetic-timescale virtual impedance adaptation with electromechanical-timescale inertia–damping coordination for PV-storage systems operating in weak grids.

### 5.4. Noise-Perturbation and Parameter-Sensitivity Analysis

To further verify the measurement-noise robustness of the proposed transient frequency sensing strategy, an additional noise-perturbation simulation is conducted. In this test, the system is assumed to operate under a weak-grid electromechanical disturbance scenario. A load-side disturbance is introduced at *t* = 0.6 s to emulate a sudden active-power imbalance, and a recovery transient is applied at *t* = 1.2 s to represent the subsequent regulation process. Meanwhile, a band-limited additive noise component is superimposed on the measured frequency-derivative signal to emulate sensor noise, derivative-estimation error, and digital sampling uncertainty. The nominal noise RMS is approximately 0.02 rad/s^2^, and the control sampling period is 100 μs, as listed in Table 2. Therefore, the test includes both actual electromechanical transients and high-frequency measurement perturbations, allowing the sensing loop to be evaluated in terms of false-trigger suppression, real-disturbance detection, and activation delay.

**Figure 21:**
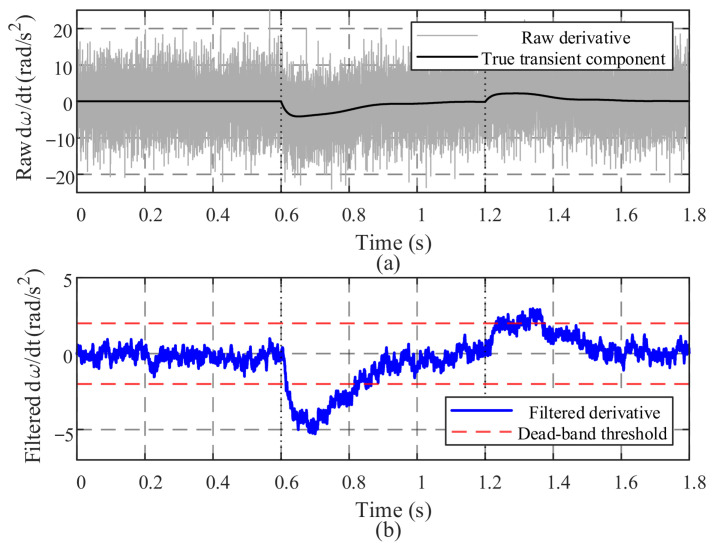
Noise-perturbation verification of the filtered frequency-derivative sensing strategy: (**a**) raw frequency derivative with measurement noise; (**b**) filtered frequency derivative with the dead-band threshold.

Figure 21 compares the raw and filtered frequency-derivative signals under the injected measurement noise. The raw frequency derivative contains high-frequency fluctuations that may repeatedly cross the adaptive-control activation boundary if directly used for parameter updating. After the proposed LPF and dead-band logic are applied, the filtered derivative remains within the dead-band during quasi-steady intervals, thereby preventing unnecessary high-frequency modulation of the adaptive *J*-*D* loop. At the same time, the transient events around *t* = 0.6 s and *t* = 1.2 s are still detected effectively, indicating that the proposed sensing strategy suppresses noise-induced false triggering without completely sacrificing transient sensitivity.

**Figure 22:**
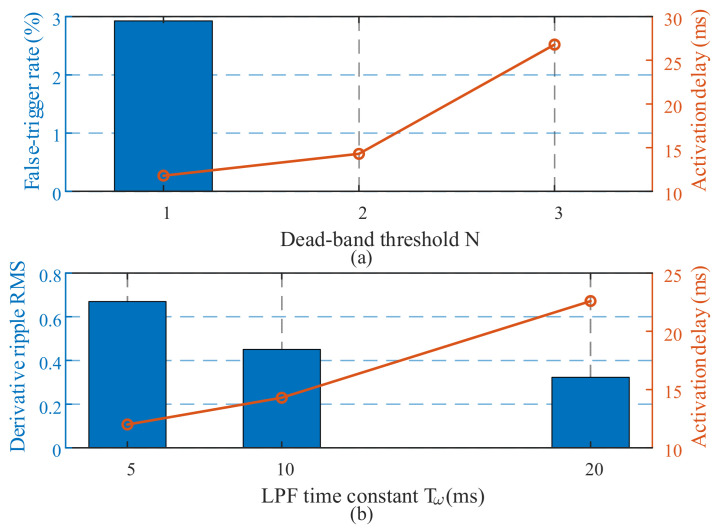
Parameter-sensitivity analysis of the frequency-derivative sensing loop: (**a**) effect of the dead-band threshold N on false-trigger rate and activation delay; (**b**) effect of the LPF time constant $T_{\omega }$ on derivative ripple RMS and activation delay.

Figure 22 further evaluates the sensitivity of the sensing loop to the dead-band threshold *N* and the LPF time constant *T_ω_*. When *N* is too small, the sensing loop becomes more sensitive to residual derivative noise, which increases the false-trigger probability. Increasing *N* improves noise immunity, but it also increases the activation delay because a larger transient derivative is required to trigger the adaptive update. Similarly, a smaller *T_ω_* provides a faster response but allows a more high-frequency derivative ripple to pass through, whereas a larger *T_ω_* improves ripple suppression at the cost of a longer delay. These results confirm the trade-off between measurement-noise rejection and transient response speed.

Based on the sensitivity results, *N* = 2.0 rad/s^2^ and *T_ω_* = 10 ms are selected as the nominal parameters in this study. Under this setting, the proposed LPF + dead-band strategy maintains sufficient sensitivity to real electromechanical transients while reducing noise-induced false triggering. As summarized in Table 4, the false-trigger rate decreases from approximately 3.0% at *N* = 1 rad/s^2^ to approximately zero at *N* = 2 rad/s^2^ and *N* = 3 rad/s^2^, while the corresponding activation delay increases from about 11 ms to 14 ms and 28 ms, respectively. For the LPF sensitivity test, increasing *T_ω_* from 5 ms to 20 ms reduces the derivative ripple RMS from approximately 0.67 rad/s^2^ to 0.32 rad/s^2^ while increasing the activation delay from approximately 12 ms to 23 ms. These quantitative results support the selected sensing parameters and demonstrate that the proposed strategy provides a practical compromise between noise immunity and dynamic response speed.

It should be noted that the present noise-perturbation test is still simulation-based. Practical sensor quantization, ADC delay, communication delay, and converter non-idealities may further influence the measured frequency derivative. These implementation factors are briefly acknowledged at the end of Section 5.5 and further discussed as future work in the final paragraph of the Conclusions.

### 5.5. Quantitative Summary of Comparative Simulation Results

To provide direct quantitative evidence for comparative validation, Table 4 summarizes the key performance indicators extracted from Figure 9, Figure 10, Figure 11, Figure 12, Figure 13, Figure 14, Figure 15, Figure 16, Figure 17, Figure 18, Figure 19, Figure 20, Figure 21 and Figure 22. The selected metrics include DC-link voltage regulation, active-power response, frequency deviation, output-current peak, voltage-sag response, transient inrush current, and noise-resilient frequency-derivative sensing. In addition, the battery-current stress indicators under the irradiance-step test are separately quantified in Table 3. Approximate values are reported when indicators are estimated from waveform plots.

The comparison cases in Table 4 are selected to represent three typical control categories. The fixed-parameter VSG represents conventional grid-forming control without adaptive transient-state sensing. The single-function adaptive strategies represent either electromechanical-timescale *J*, *D* regulation or electromagnetic-timescale *L*_vir_ regulation. In contrast, the proposed strategy combines adaptive *J*, *D*, adaptive *L*_vir_, HESS power decoupling, and LPF + dead-band-based noise-resilient sensing under the same disturbance conditions.

As shown in Table 4, the proposed strategy improves the main dynamic indicators compared with the fixed-parameter and single-function adaptive strategies. The maximum frequency deviation is reduced from approximately 0.6 Hz to 0.15 Hz, and the transient inrush-current peak is reduced from approximately 620 A to 290 A. The output-current peak is also reduced from more than 1.5 p.u. to approximately 1.15 p.u. In addition, the noise-perturbation results show that increasing *N* and *T_ω_* improves noise immunity but introduces additional activation delay, confirming the trade-off between sensing robustness and response speed.

Overall, Table 3 and Table 4 complement the waveform-based analyses in Section 5.1, Section 5.2, Section 5.3 and Section 5.4. Table 3 provides direct battery-current stress indicators for the HESS power-decoupling mechanism, while Table 4 summarizes the comparative dynamic performance of the proposed multi-timescale sensing framework against the selected baseline and ablation strategies.

It should be noted that the present validation is still based on a single-node PV-HESS weak-grid configuration. Although the proposed strategy shows improved performance under the tested irradiance-step, load-disturbance, voltage-sag, and measurement-noise conditions, its scalability under multi-node distribution networks, different PV-storage allocation scenarios, unbalanced faults, harmonic-rich grid conditions, and practical hardware non-idealities still requires further investigation. In addition, the battery-stress evaluation in this work is based on current-related indicators, including RMS current, high-frequency current RMS, peak current, and charge throughput, rather than a complete electrochemical aging model. Therefore, the reported battery-related results should be interpreted as current-stress reduction evidence under the tested condition.

## 6. Conclusions

To improve the transient resilience of PV-storage systems operating in weak grids, this paper proposes a multi-timescale transient-state sensing and signal-processing framework that integrates HESS power decoupling, voltage-deviation-driven adaptive virtual inductance, and noise-resilient frequency-derivative sensing. The main conclusions are summarized as follows:

(1) By introducing frequency-domain HESS power decoupling, high-frequency transient power components are mainly assigned to the supercapacitor, while the battery handles slower energy variations. The added battery-current stress evaluation shows that the proposed HESS decoupling reduces the high-frequency battery-current RMS by 50.8% and the battery peak current by 59.5% under the tested irradiance-step condition. These results indicate that the proposed strategy can reduce measurable battery-current stress and may help mitigate micro-cycling tendency, although a complete electrochemical lifetime prediction requires further aging-model or experimental validation.
(2) A voltage-deviation-driven adaptive virtual inductance strategy is introduced to improve transient current suppression during voltage-sag events. Under the tested voltage-sag condition, the proposed strategy reduces the peak inrush current from approximately 620 A to 290 A, corresponding to a 53.2% reduction. This result indicates improved current-limiting performance under the specified simulation condition, without implying hardware survivability under all possible fault scenarios.
(3) A noise-resilient frequency-derivative sensing strategy was designed to improve adaptive inertia and damping control under measurement noise. By combining a first-order LPF with a dead-band for false-trigger suppression, the proposed sensing logic reduces noise-induced false triggering while preserving sensitivity to real transient events.

The simulation results indicate that combining transient-state sensing, adaptive impedance shaping, and HESS power decoupling can provide an effective and practically meaningful approach for enhancing the resilience of grid-forming renewable energy systems under the tested weak-grid conditions. Future work will extend the proposed strategy to multi-node benchmark distribution networks, such as IEEE 33-bus and IEEE 69-bus systems, to evaluate its scalability under different PV-storage allocation scenarios and grid-strength conditions. Hardware-in-the-loop and experimental verification will also be conducted to further examine the effects of sensor quantization, digital delay, converter nonlinearities, grid-impedance variation, and harmonic interactions on the proposed sensing and control framework.

## Figures and Tables

**Table 1:** Main electrical and control parameters.

Parameters	Values	Parameters	Values
*U*_dc_/V	700	*ω*_0_/(rad·s^−1^)	314
*U*_g_/V	380	*J*_0_/(kg·m^2^)	0.8
*f*/Hz	50	*D*_0_/(N·m·s·rad^−1^)	50
*R*_f_/Ω	0.1	*k*_p_	1200
*L*_f_/mH	1.7	*k*_1_	1.8
*C*_f_/μF	22	*k*_2_	1.6
*R*_g_/Ω	0.1	*α*	1.5
*L*_g_/mH	2.1	*β*	0.8
*S*/(W/m^2^)	1000	*T*/°C	25
*L*_0_/mH	1.0	*N*/(rad·s^2^)	2.0
*f*_sw_/kHz	10	*k*_vir_	0.005
*C*_dc_/μF	2200	*λ*	0.05
*P*_pv_/kW	10	*T_ω_*/ms	10
*P*_ref_/kW	10	*T_f_*/ms	20

**Table 2:** Supplementary simulation settings and comparison cases.

Item	Setting	Item	Setting
Simulation platform	MATLAB R2024b/Simulink	Solver type	Fixed-step discrete
Simulation step size	1 μs	Control sampling period	100 μs
Switching frequency	10 kHz	Total simulation time	2.0 s
Inverter rated power	15 kVA	Rated frequency	50 Hz
Rated AC voltage	380 V	DC-link reference voltage	700 V
Battery rated voltage	500 V	Battery rated capacity	40 Ah
Battery initial SOC	80%	Supercapacitor capacitance	20 F
Supercapacitor rated voltage	500 V	Supercapacitor initial voltage	400 V
Irradiance-step test	1000 → 1400 → 600 W/m^2^	Grid-strength test	SCR = 20 and SCR = 2.5
Load disturbance	+2 kW at *t* = 0.6 s	Power-reference change	10 kW → 8 kW at *t* = 1.2 s
Voltage-sag test	Three-phase symmetrical sag	Sag duration	*t* = 0.6–1.2 s
PCC voltage change	311 V → 270 V	Voltage-sag depth	13.2%
Dead-band thresholds tested	*N* = 1, 2, 3	LPF time constants tested	*T_ω_* = 5, 10, 20 ms
Fixed-parameter VSG	*J* = *J*_0_, *D* = *D*_0_, and *L*_vir_ = *L*_0_	Adaptive *J*, *D* only	Adaptive *J*, *D*, *L*_vir_ = *L*_0_
Adaptive *L*_vir_ only	Adaptive *L*_vir_, *J* = *J*_0_, *D* = *D*_0_	Proposed strategy	Adaptive *J*, *D*, adaptive *L*_vir_, HESS decoupling, LPF + dead-band

**Table 3:** Battery-current stress indicators under the irradiance-step test.

Indicator	Single-Battery Compensation	Proposed HESS Decoupling	Reduction
Battery RMS current (A)	7.648	6.738	11.9%
High-frequency battery-current RMS (A)	2.393	1.176	50.8%
Battery peak current (A)	25.301	10.240	59.5%
Battery charge throughput in tested interval (mAh)	3.38	3.00	11.1%

**Table 4:** Quantitative comparison of dynamic and transient performance indicators for the fixed-parameter VSG, single-function adaptive strategies, and the proposed strategy under the tested disturbance scenarios.

Test Case	Metric	Fixed-Parameter VSG	Single-Function Adaptive Strategy	Proposed Strategy
Irradiance step	DC-link voltage overshoot	—	—	≈8 V
	DC-link recovery time	—	—	≈0.1 s
Weak grid, SCR = 2.5	Active-power overshoot	≈24%	—	<19%
	Active-power undershoot	≈31%	—	Lower and smoother
	Maximum frequency deviation	≈0.6 Hz	≈0.35/0.25 Hz	≈0.15 Hz
	Output-current peak	>1.5 p.u.	≈1.4 p.u.	≈1.15 p.u.
Voltage-sag test	PCC voltage during sag	311 → 270 V	Same	Same
	Adaptive virtual inductance	Fixed	Partially adaptive	≈1.2 → 5.4 mH
	Transient inrush-current	≈620 A	≈580/310 A	≈290 A
Noise perturbation	False-trigger rate at N = 1/2/3 rad/s^2^	—	—	≈3.0%/0%/0%
	Activation delay at N = 1/2/3 rad/s^2^	—	—	≈11/14/28 ms
	Derivative ripple RMS(rad/s^2^) at *T_ω_* = 5/10/20 ms	—	—	≈0.67/0.45/0.32
	Activation delay at *T_ω_* = 5/10/20 ms	—	—	≈12/15/23 ms

## Data Availability

The data presented in this study are available on request from the corresponding author.
